# Can I control my bowel symptoms myself? The experience of controlling defaecation dysfunction among patients with rectal cancer after sphincter-saving surgery: a qualitative study

**DOI:** 10.1080/17482631.2022.2031832

**Published:** 2022-02-13

**Authors:** Wen Liu, Hai Ou Xia

**Affiliations:** a School of Nursing and health Management, Shanghai University of Medicine and Health Sciences, Shanghai, People’s Republic of China; b School of Nursing, Fudan University, Shanghai, People’s Republic of China

**Keywords:** Rectal cancer, defaecation dysfunction, symptom management, nursing, qualitative study

## Abstract

**Purpose:**

To explore the experience of controlling defaecation dysfunction among patients with rectal cancer after sphincter-saving surgery.

**Methods:**

This study applied a descriptive qualitative design. Thirty-six patients with rectal cancer were given semi-structured interviews in mainland China from February to July in 2019 after sphincter-saving surgery. Participants were recruited by purposive sampling. The thematic analysis approach was applied to analyse the transcripts.

**Results:**

Three major themes emerged from the data were “having motivations of controlling defecation dysfunction”, “using strategies of controlling defecation dysfunction” and “facing barriers of controlling defecation dysfunction”.

**Conclusion:**

Defaecation dysfunction makes obvious problems for patients after sphincter-saving surgery, although patients tried some self-care methods to cope with the defaecation dysfunction, some barriers still exist in the process of self-controlling of bowel symptoms. There is a strong demand for a systematic and scientific guideline for the self-management of defaecation dysfunction.

## 1. Introduction

Colorectal cancer is one of the most common malignant tumours in the world.(Bray et al.,^2018^) In China, the lifestyle of people has changed a lot under the rapid development of the economy, and an increasing number of people are consuming high-sugar and high-fat foods, and individuals are more likely to be exposed to environmental contamination than before. As a result, China is surpassing other nations in both the number of people who are diagnosed with colorectal cancer and the number of people who die from colorectal cancer. (Ferlay et al.,^2015^) According to the latest global cancer data, there were 5.5 million new cases of colorectal cancer in China in 2020, and 2.8 million colorectal patients died that year.(Sung et al.,^2021^)

Due to advances in surgical technology, traditional abdominal perineal resection (APR) is gradually being applied instead of low anterior resection (LAR), which could preserve the anus. Sphincter-saving surgery is considered the first choice for patients with middle-low rectal cancer. (Saito et al.,^2011^; Sauer et al.,^2004^) However, various bowel symptoms emerge after sphincter-saving surgery, such as varying degrees of defaecation frequency, stool incontinence, defaecation urgency, inability to fully defaecate, and defaecation strain, which are together called low anterior resection syndrome (LARS). (Juul et al.,^2014^; Ziv et al.,^2013^). It has been confirmed that up to 90% of rectal cancer survivors after surgery in Europe and North American had been suffered from these symptoms. Moreover, these symptoms, which have a strong influence on quality of life, have been increasingly confirmed that they will exit for a long time after surgery. (Keane et al.,^2017^; Reinwalds et al.,^2018^)

In recent years, an increasing number of researchers in China have started to focus on this issue, but they are still in the stage of describing the situation of defaecation dysfunction. Retrospective research has reported that the incidence of LARS is 34.8% and of severe LARS is 17.8%. (Ishiyama et al.,^2014^). Regarding the duration of bowel symptoms, several studies have noted that it takes at least six months for the dysfunction to be relieved, which is consistent with the results reported in other countries. (Pu. Yu,^2017^; Xu et al.,^2011^; Yan,^2014^). Since there is scant evidence on the condition of defaecation dysfunction more than a year after surgery, the duration of this symptom among Chinese patients is not clear.

Although these bowel symptoms are common, there are few mature therapies available to control them. The treatments reported in studies in China for controlling defaecation dysfunction mainly include biological feedback, anal douche, sacral nerve stimulation, anal sphincter training and traditional Chinese medicine(Rosen H et al,^2011^; Thomas GP et al,^2015^). A consensus regarding these treatment methods has not been reached, and the effectiveness of some of them needs to be further confirmed. Moreover, these methods have not been popularly implemented in hospitals. As a result, the control of bowel symptoms typically comes down to the self-management of patients, which involves the adjustment of diet, social activities and mindset and the use of certain drugs.(Landers et al.,^2012^)

On the whole, evidence on bowel symptom management and its effectiveness has been described in only a few studies. Margaret et al conducted a review and reported that the range of self-care strategies used by patients to manage bowel symptoms can be grouped under three main themes: functional self-care strategies, social activity-related self-care strategies, and alternative self-care strategies. Functional self-care strategies focus on managing bowel movements. These strategies include diet and fluid modification, bowel medication use and the wearing of protective pads or clothing. A nursing intervention guided by self-management materials and drawn up by the faculty of nursing at the University of California was given to patients three months after surgery. The materials included information on disease, emotion and role management. It was found that this intervention can effectively improve patients’ psychological status and ability to cope with defaecation dysfunction (Z. Yu,^2015^) In China, research focusing on the self-management of patients after surgery is in the initial stages, although the number of studies in this field is increasing. The research concentrates primarily on describing the status of bowel symptoms, and few studies have deeply explored specific methods for managing bowel symptoms.

Therefore, this study explored the experience of controlling defaecation dysfunction among patients with rectal cancer following sphincter-saving surgery. We aimed to further identify the barriers that exist in the management of bowl symptoms, providing evidence that doctors and nurses can use to adopt effective measures and promote the quality of life of rectal cancer survivors.

## 2. Methods

### 2.1 The study

This research used a descriptive qualitative design to explore the experience of controlling defaecation dysfunction among patients with rectal cancer and to understand how patients cope with these symptoms.

### 2.2 The participants

Eligible participants were outpatients who had been diagnosed with rectal cancer and had undergone a sphincter-saving operation between 2 months and 2 years before recruitment. The participants did not have any hearing or cognitive disorders and were able to agree to or withdraw from the study. Patients with complications (anastomotic fistula, etc.) after surgery or patients having suffered from some illness before surgery, such as inflammatory bowel disease and irritable bowel syndrome were excluded. Patients who had always taken medication that affects bowel function and those whose medical records were not complete were also excluded.

Participants were recruited by purposive sampling. The lead investigator explained the aim of the study to participants and told them that they could refuse to join or withdraw at any time. Subsequently, the participants who volunteered to participate in the study signed an informed consent form. Thirty-six patients diagnosed with rectal cancer who visited the clinic for follow-up treatment after sphincter-saving surgery at a general hospital were included in this research. Participants were represented by an alphanumeric code, where C = participant. The participants’ demographic information is given in Table I.

**Table I.** Demographic and treatment characteristics of participants

Characteristics	n	%
Age(years)		
≤55	7	19.4
(56,65]	15	41.7
(66–75]	11	30.6
≥76	3	8.3
Gender		
Male	24	66.7
Female	12	33.3
Marital status		
Married	22	61.1
Divorced	10	27.8
Widower	4	11.1
Education		
Less than junior school	8	22.2
Middle and high school	19	52.8
College graduate and higher	9	25.0
Occupation On- job	13	36.1
Retirement	23	63.9
Economici status of family		
Poverty	3	8.3
Simply having adequate food and clothing Well-off Rich	9 20 4	25 55.6 11.1
Time from surgery to interview(months)		
(0,3]	6	16.7
(3,6]	9	25
(6,12]	12	33.3
(12,24]	9	25
Type of operation		
Robotic anterior resection of rectal cancer	28	77.8
3D laparoscopic assisted anterior rectal resection	8	2.2
Distance between the anal margin and the lower margin of tumour(cm)		
(2,5]	6	16.7
(5,10]	19	52.8
(10,20]	11	30.5

### 2.3 Setting

All the interviews were conducted in a quiet, private room at the hospital, in the patient’s home or at some other place the patient thought convenient. During the interview process, only the researcher and the patient were present, and the first author(WL) conducted all the interviews. All interviews were conducted from March to July 2019.

### 2.4 Data collection

Demographic information was collected before the interview. During March and July 2019, the first author contacted the participant either face-to-face in the outpatient or by telephone to set up a convenient location and time for the interview and then conducted the in-depth interview using a semi-structured interview guide (Table II). The interview guide was developed according to symptom management theory (SMT), which includes three related elements: experience of the symptoms, symptom management strategies and symptom outcomes. (Dodd et al.,^2001^) Interviews were conducted one to two times for each participant, lasting 25 to 80 minutes for each interview. If a participant’s data from the first interview were found to be incomplete or confusion about the data arose during the primary analysis process, the first author would contact the participant for the second interview. All interviews were audio-recorded and field notes were made during the interviews.Table II presents the interview guide.

**Table II.** Interview guide

Interview questions
Is there any bowel symptoms existing after surgery? What are the most distressing bowel symptoms? How the symptom influence on your daily life? How do you cope with the defaecation dysfunction? What factors do you think could influence the bowel symptoms? How do you deal with these factors? How do you find the methods to manage the bowel symptoms? Do you think these bowel symptoms could be relieved by your management? Who do you want most to give you suggestions and assistance about managing bowel symptom and why? What do you think is the most difficult part in managing these symptoms and could you describe it in detail?

### 2.5 Ethics consideration

Ethical approval of this study has been granted by the Institutional Review Boards of University, school of nursing(#TYSQ2019-6-01). All the participants joined this study voluntarily, and they could withdraw from the study at any time. All the data collected from the participants were kept by a designated person and were treated as confidential.

### 2.6 Data analysis

Data collection and analysis were conducted simultaneously. The interviews were transcribed verbatim by the interviewer within 24 h after each interview. The data analysis process was based on the principles of thematic analysis outlined by Braun and Clarke (Braun & Clarke,^2006^). Interview transcripts were uploaded into NVivo11.0 to facilitate data coding.

The steps of the analysis were as follows: (1) the transcripts from interviews were read several times to get a general and sufficient understanding of the data. The reading process was performed independently by two researchers(WL, HJ) (2) Meaning units (sentences or paragraphs) corresponding to the participants’ experiences of controlling defaecation dysfunction were selected using an inductive approach;(3) Two researchers(WL,HJ)coded the transcripts independently. Then the third researcher (HO) checked for discrepancies between the two coding results and discussed them with these two researchers, and the final codes agreed (4)The codes were firstly grouped into subthemes according to their similarities and differences;(5)then the subthemes were grouped into themes.

### 2.7 Rigour enhancing

The rigour of this study was upheld by the following methods:

First, the interviewer was well prepared before the interviews. The interviewer was a doctoral nursing student with past experience with and firm mastery of qualitative research. Furthermore, the interviewer had spent 6 months in the colorectal ward and outpatient clinic to become familiar with the clinical background of the participants, helping the interviewer acclimatize to the interview situation.

Second, during the interview, the researchers communicated with the participants in a relaxed environment and listened closely to the experience of the participants in a neutral and uncritical manner.

Third, after the interview, the researcher called the participant back if there was some uncertainty or unclear information in the interview data so that the data obtained from each participant would be accurate.

Fourth, the initial codes and categories, and especially the discrepancies between the team members, were discussed among the authors until a consensus was reached.

Fifth, repeated reading of the transcripts and writing of reflections and a researcher with considerable qualitative research experience verified the data analysis and the results.

## 3. Results

Totally 36 patients diagnosed with rectal cancer after sphincter-saving surgery were interviewed in this study. Three major themes with 13 corresponding subthemes emerged from the data (Table III).

**Table III.** The themes and subthemes generated from the results

Theme	subtheme	Selected quotes
Having motivation to control defaecation dysfunction	Driven by the physical burden of defaecation dysfunction	“I had to defecate once an hour before, and it later became better, once every 2 hours. 1am,2am,3am … that was the time I should get up for defecation. Of course, sleeping for a whole night was almost impossible for me, so I had to find all the possible times to go to sleep at daytime” “the first two months were really miserable to me that I can hardly control a little.When I felt to defecate and I stand up from the bed,at the same time, the stool is leaking out of the anus and the trousers were polluted at once.” “Absolutely the defecation got better, but ‘normality and health’ was far away from me. I was a little anxious to determine the effective methods to control abnormal defecation.”
	Driven by the physiological burden of defaecation dysfunction	“I have none of these functions now, so I have to make efforts to modulate the status, but I think the modulation will be a long-term project.” “15 months passed and the symptom is still existing … … Could you please tell me how long my defecation could return to normality as the status before surgery? Or it won’t return forever … … ” “It was embarrassed that I suddenly got the feeling of defecation at meal with my friends and I had no idea how to explain to them because I can hardly wait a minute in case that the stool may leak out at anytime, and that’s what I were the most ashamed of. I can’t image if the stool leak out in front of my friends ….” “Once I farted, and there was definitely some excrement coming out as well. I was scared about this, so I would rather hold my flatus for a while.” “I would go away from the crowd once I wanted to fart, or others might feel strange and wonder, ‘where did the foul smell come from’. In order to decrease the flatus I tried to reduce the amount of some foods in my diet.”
	Driven by the social burden of defaecation dysfunction	“I dared not to take part in the dinner with my friends and relatives and I was worried about whether I could find out the toilet once I want to defecate.” “I don’t need my friends to my home to visit me,specifically,I don’t want to see anyone else. For 1 hour’s talking,I may go to the toilet several times and that’s embarrassing.” “I went to the Disneyland with my grandson and everything seemed normal that day. However, the day after that day it turned worse suddenly so I guess if I have eaten something bad or unfit for my bowel in Disneyland. Seems that I still can’t go out for more activities.” “Her trousers and the sheet were contaminated everywhere by the stool that night. When I finished washing the sheet and came back to bedroom, the stool was everywhere again! I knew it was the bamboo shoots that caused the loss of control of defecation so I advised her not to take it anymore in the future.”
Using strategies to control defaecation dysfunction	Obtaining information support from others	“When I returned to the clinic for examinations after surgery, I asked the doctor how to cope with the bowel symptoms, and he told me that I could try to practice contracting the levator ani muscle.” “I asked him why my flatus was so much everyday and Doctor Xu told me that it was attributed to two much meat in my diet. So I came back home to lessen it.” “my workmate also suffered from this disease years ago, he told me that it was better to form the regular defecating habit-to defecate once at morning, once at noon and once evening. I am still making efforts in this way.” “some suggestions are given by my son and he found them on internet or somewhere else. Different fruits and organic vegetables were always sent to my house everyday which were ordered in the internet by him.” “I had always been bearing the symptoms of defecating nearly 10 times a day, and I had to find help from Chinese medicine. It has decreased to 4–5 times now, I guess that may be related to the Chinese medicine that I have taken within these two months.” “My wife cut the whole apple into pieces and made them easier for me to eat nearly every day; sometimes she even squeezed the fruits into juice for me. You know, fruit was not my preference in the past.”
	Dietary modification	“I have found that too much food with starch worsens the bowel symptoms; for example, noodles and sweet potato in particular led to much more flatus, and the stool leaked out of the anus with the flatus”. “Watermelon was found to aggravate symptoms on several occasions, especially in summer, so I shouldn’t eat too much”. “I thought banana and Chinese chives were helpful to me, as they made the defecation be smoother and easier for me”.
	Application of assistive medications and tools	“I put some tissues or pads under the ass just at night in case that bed and trousers were made dirty during sleep”.(C15) “when I go outside, I prepare some tissues on the underpants, like this tissue that I folded it at home.” “I had taken probiotics for one or two years before surgery, and it was helpful; now, the probiotics also help a lot when the stool is hard to defecate.” “sometimes I wanted to defecate, but however I make efforts it just wasn’t discharged … … I used to take probiotics when this situation occurred before and it really worked so I tried this medicine now.”
	Readaptation of the new defaecating habit	“I thought there was no need to moderate the defecation because in my opinion that was not illness but normality. We always said ‘once the belly has been cut, 10 years will be needed to repair it’. I thought there was no need to worry about it, and it is indeed recovering now.” “I tried and knew that there was a great chance to defecate meanwhile with the meal, so I just needed to discharge the stool before the meal and that situation wouldn’t happen any more.”
	Choosing activities helpful for controlling bowel symptoms	“That was really a little strange, I have found out that when I was working outside, the feeling of defecation never came to me, and if I spent the same time staying at home, I would have gone to the toilet several times. So I preferred to spend more time outside!” “Sometimes when I finished walking outside and went back home, the difficulties in defecating went away, and the stool could be discharged smoothly. Walking made defecation comfortable!” “I occasionally found out that after I finished exercising with certain equipment, the defecation became extraordinarily smooth that day, and only that particular equipment worked like this!” “I always found that when I kept the sitting position, the feeling of urgent defecation was lessened and I could control it. But once I stood up, I had to go to the toilet as soon as possible. So I knew I could drive a car to work by myself and that would be safer than taking bus or walking.” “once I started to walk, the stool came quickly and couldn’t be held on. So I just walked near the house.”
Facing barriers to controlling defaecation dysfunction	Confusion arising from conflicting information	“I asked the doctor how to manage the bowel symptoms, and he just told me there was no need to pay much attention to manage them because these symptoms would recover as time went by. But many other patients tried to control them by themselves and some methods seemed work. What should I do?”
	Overreliance on personal intuition	“Although doctors told me that foods rich in fiber were good for smooth defecation, like corn and celery, I thought these foods were so stiff that they may hurt my bowel or they may damage the wound in my bowel. I have never eaten them during these months.” “The old people in our family always told us the Chinese chives were stimulating food and that was also the rule from our old ancestors. I believed it without any suspicion, and it was forbidden in my diet.”
	Confusion caused by the different requirements for treatment and controlling defaecation dysfunction	“The more food I ate, the more frequent would the defecation be, and the anus would be more painful, but I also knew if I didn’t eat enough food then I couldn’t stand the chemotherapy any more. That would be fatal to me. That would be not good for my recovery. So I told the doctor once the chemotherapy was over, I won’t eat so much food anymore”
	Lack of self-discipline	“doctors told me to eat more vegetables and fruits in my daily life, I tried my best to do that at the beginning after surgery, but I really don’t like vegetables, so I gradually quit nowadays.”
	Constraints related to objective social and family factors	“Recently, I was taking meals at my relative’s home, and she prepared many dishes including too much meat. I couldn’t blame her, but I did not want to eat so much greasy food in case it caused more frequent defecation.” “my workmates often ordered so much dishes that I had eaten too much food unconsciously. I found that once I took meal with workmates at restaurant, I had to defecate more frequently the next day.”

### 3.1 Having motivation to control defaecation dysfunction

Defaecation dysfunction was an obvious burden to participants’ daily lives after sphincter-saving surgery, which pushed them to control it as much as possible. The management of defaecation dysfunction was driven by three factors: the physical burden, the psychological burden and the social burden of defaecation dysfunction.

#### 3.1.1 Driven by the physical burden of defaecation dysfunction

Bowel symptoms, including faecal incontinence, increased frequency of defaecation, abnormal sensations associated with defaecation, irregular bowel movements, constipation, morphological changes in stool, abnormal flatus and so on, were noted among all the participants in the interviews. Uncomfortable feelings, such as pain, fatigue, and abdominal distension, could be caused by these symptoms, which were an obvious physical burden on participants and affected their quality of life.

Some participants reported that the increased frequency of defaecation caused complications such as pain around the anus, and the pain was so strong that some participants felt it was unbearable, forcing them to search for methods to control their frequent defaecation. For example, one participant said, “I can’t bear anymore, I just went to the toilet time and time again every day, nearly 20 times or more, causing severe pain around my anus. Once I went to the toilet, the pain became more severe! I just thought I was going to die! I was always finding methods to avoid too much defecation!” (C13)

Having to defaecate at night was reported by most participants. Some participants said they had to get up to defaecate every 1 to 2 hours; moreover, some of them reported that they could not sleep the whole night due to too many defecations, which made them feel tired during the daytime. “I had to defecate once an hour before, and it later became better, once every 2 hours. 1 am, 2 am, 3 am … that was the time I would get up to defecate. Of course, sleeping for a whole night was almost impossible for me, so I had to find all the possible times to sleep in the daytime” (C24).

Over time, the overall physical burden of bowel symptoms showed a decreasing trend. The frequency of defaecation decreased, the stool became thicker and longer, and the ability to control defaecation recovered. However, nearly half of the participants reported that there was still something wrong with their defaecation at least 15 months after the operation, which made them search for ways to return to normal defaecation as soon as possible. “Absolutely the defecation got better, but ‘normality and health’ was far away. I was a little anxious to determine the effective methods to control abnormal defecation” (C19).

#### 3.1.2 Driven by the physiological burden of defaecation dysfunction

The defaecation function of participants changed substantially after surgery, causing their lives to fall into disorder and creating various psychological burdens. This was another reason forcing participants to control their defaecation dysfunction.

When participants sensed the functional absence of the bowel and anus, they felt considerable anxiety about whether and when the function would return, and they were eager to find solutions to this problem. “I have none of these functions now, so I have to make efforts to modulate the status, but I think the modulation will be a long-term project” (C22).

Participants showed obvious uncertainty because of their sense of loss of control, especially when they found that no method was effective in controlling their defaecation dysfunction, and this feeling became stronger as time went by. “Fifteen months passed and the symptoms were still present … could you please tell me how long it will be until [my defecation] will return to normal, like it was before surgery? I have tried several ways to control it, but they didn’t work. Are there any more methods to control the bowel symptoms?” (C2)

Some participants adopted strategies to avoid the embarrassment caused by abnormal defaecation, including being unable to hold the defaecation, stool coming out together with flatus and smelly flatus. These symptoms caused the participants to feel ashamed, especially when they were with other people. One participant said, “Once I farted, and there was definitely some excrement coming out as well. I was scared about this, so I would rather hold my flatus for a while” (C19). Another participant said, “I would go away from the crowd when I wanted to fart, or others might feel uncomfortable and wonder, ‘Where did the foul smell come from?’ In order to decrease the flatus, I tried to reduce the amount of some foods in my diet” (C33).

#### 3.1.3 Driven by the social burden of defaecation dysfunction

Defaecation dysfunction also imposed a social burden in participants’ daily lives, and to engage in certain social activities, they used various methods to find the balance between defaecation dysfunction and social activities.

Not being able to stop the stool from leaking led some participants to refuse to join dinner parties or gatherings organized by their friends because they were afraid of “making a fool of themselves”. “I did not dare take part in dinners with my friends and relatives, as I was worried about whether I could find the toilet when I wanted to defecate” (C7).

Frequent urgent defaecation, which was found more often in participants who were less than 6 months post-surgery, was reported to interrupt normal social activities. To make social activities go smoothly, some participants chose to take action to control their defaecation. One participant said, “If I knew I was going out to a re-examination in the clinic like this afternoon, I would cut down the amount of food at lunch in case the frequent urgent defecation emerged when I was having consultation with the doctor” (C32).

Abnormal defaecation caused considerable trouble not only in participants’ daily lives but also in their family members’ lives. Sometimes, defaecation dysfunction made their lives complicated, and family members were responsible for cleaning up messes. This pushed patients’ family members to assist in managing the bowel symptoms and adapt to the new life of the patients. “Her trousers and the sheet were contaminated everywhere by the stool that night. When I finished washing the sheet and came back to the bedroom, the stool was everywhere again! I knew it was the bamboo shoots that caused the loss of control of defecation, so I advised her not to eat them anymore in the future” (C24).

### 3.2 Using strategies of controlling defaecation dysfunction

The participants tried various methods to control their defaecation dysfunction, and these methods were applied with the support of medical instructors, family members and other social relations.

#### 3.2.1 Obtaining information support from others

Participants sought help from others to control their bowel symptoms; most of them mentioned that they had asked the doctor how to relieve their defaecation dysfunction, and instructions from doctors were provided mostly at the clinic. For example, a participant said, “When I returned to the clinic for examinations after surgery, I asked the doctor how to cope with the bowel symptoms, and he told me that I could try to practice contracting the levator ani muscle” (C2).

Chinese medicine is a common method of treatment aiding in the recovery of patients with cancer. Several participants tried to find Chinese medicine-based solutions to relieve their bowel symptoms, but they also said that they were not sure the improvement in their defaecation function was definitely due to the Chinese medicine. “I had always been bearing the symptoms of defecating nearly 10 times a day, and I had to find help from Chinese medicine. It has decreased to 4–5 times now, I guess that may be related to the Chinese medicine that I have taken these last two months” (C10).

Participants reported that they also preferred to talk to other patients who had the same problem. This helped them find support to understand their symptoms, face the difficulties and manage the dysfunction. Even though they did not know whether these suggestions were useful, they truly wanted to try them. One participant said, “My workmate also suffered from this disease years ago; he told me that it was better to form a regular defecating habit, namely, to defecate once in the morning, once at noon and once in the evening. I am still making an effort to do this” (C11).

Experiencing defaecation dysfunction led the participants to rely more on their family members as well. Family members tried to assist the patient in coping with the dysfunction by persuading patients to maintain a structured life pattern. “My wife cut whole apples into pieces and made them easier for me to eat nearly every day; sometimes she even squeezed the fruits into juice for me. You know, fruit was not my preference in the past” (C1). Regardless of whether the methods devised by the family members were correct, they seemed to be important to the participants.

#### 3.2.2 Dietary modification

Among the 36 participants, 23 confirmed that diet—both the type and amount of food—influenced their defaecation after surgery. Therefore, moderation was exercised to avoid the aggravation of defaecation dysfunction caused by diet.

The types of food that could aggravate the symptoms included greasy foods, foods high in fibre, liquid diets, gas-inducing foods, sweet foods and so on. Eight participants noted that greasy foods worsened their dysfunction. “Some greasy food, like pig’s feet and bouilli, made the defecation more frequent, so I tried to avoid those foods” (C13).

Six participants also reported that gas-inducing foods lead to more flatus, which might be combined with stool, especially during the first six months after the operation. “I have found that too much food with starch worsens the bowel symptoms; for example, noodles and sweet potato in particular led to much more flatus, and the stool leaked out of the anus with the flatus” (C25).

Two participants reported that sweet foods, such as honey, could make the incontinence worse, while two other participants said that liquid foods, such as fish soup, could increase the frequency of defaecation.

Participants reported different effects of high-fibre foods. Some of them said fibre was helpful and made defaecation smoother. In contrast, others found that some fruits and vegetables made defaecation more frequent, including watermelon, kiwi berry and green vegetables. One participant said, “Watermelon was found to aggravate symptoms on several occasions, especially in summer, so I shouldn’t eat too much” (C14). Another participant said, “I thought banana and Chinese chives were helpful to me, as they made defecation smoother and easier for me” (C26).

#### 3.2.3 Application of assistive medications and tools

Participants noted that there was a risk of defaecating anywhere at any time, especially during the first 3 months after surgery. Some participants reported that they used a hygiene pad or tissue to prevent their underpants from being contaminated by the stool. These tools seemed to be supplementary methods of managing defaecation. Participants preferred to use such tools only while sleeping or when going outside. It was also necessary to use these assistive tools during the radiotherapy process.

“When I went outside, I put some tissues on the underpants, like this tissue that I folded at home before I went outside” (C36).

In addition to the tools, assistive medications were also used by some participants according to their common sense. Probiotics that were used as a usual medicine for constipation before surgery were also chosen to relieve the difficulties in defaecating experienced after surgery.

“I had taken probiotics for one or two years before surgery, and it was helpful; now, the probiotics also help a lot when it is hard to defecate” (C4).

#### 3.2.4 Readaptation of the new defaecating habit

The interviews indicated that participants could reduce the influence of defaecation dysfunction on their lives by adapting to the new bowel habit. Their attitudes towards their bowel symptoms could determine whether they adapted to them. Active thinking seemed to be a helpful way to cope with defaecation dysfunction, at least in terms of psychological management. Several participants reported that they regarded defaecation dysfunction as normal in the process of recovery, so they showed an active attitude towards it.

“I thought there was no need to moderate the defecation because in my opinion that was not illness but normality. We always said,‘Once the belly has been cut, 10 years will be needed to repair it’. I thought there was no need to worry about it, and it is indeed recovering now” (C19).

As time went by, readaptation made participants more rational in facing their symptoms and helped them return to normal life more quickly. Several participants who were at least one year post-surgery stated that they had gotten used to the defaecation pattern even though it had not returned to normal. “I found nothing abnormal in about 3–4 defecations a day. I had gotten used to it, and no trouble was caused by it anymore” (C19).

#### 3.2.5 Choosing activities helpful for controlling bowel symptoms

Distraction was reported to be a useful method for controlling defaecation dysfunction among some participants, who found fully diverting their attention from the defaecation and occupying themselves with other activities to be useful. Four participants said that when they went out to shop or visit the clinic, the bowel symptoms were better than when they were at home.

“That was really a little strange; I found that when I was working outside, the feeling of defecation never came to me, and if I spent the same time at home, I would have gone to the toilet several times. So I preferred to spend more time outside!” (C9)

Forming a suitable pattern of exercise was also another useful method. Some participants reported that regular walking helped ease their defaecation. For example, a participant said, “Sometimes when I finished walking outside and went back home, the difficulties in defecating went away, and the stool could be discharged smoothly. Walking made defecation comfortable!” (C30) Once participants found a form of exercise that worked for managing their bowel symptoms, they were more likely to select this kind of exercise in the following days. One participant said, “I occasionally saw that after I finished exercising with certain equipment, the defecation became extraordinarily smooth that day, and only that particular equipment worked like this!” (C35)

Body positions were also reported to influence defaecation. Some participants adopted a posture that was able to relieve defaecation dysfunction. One participant said, “I always found that when I kept the sitting position, the feeling of urgent defecation was reduced and I could control it. But once I stood up, I had to go to the toilet as soon as possible. So I knew I could drive a car to work by myself and that would be safer than taking a bus or walking” (C21).

### 3.3 Facing barriers to controlling defaecation dysfunction

Some difficulties emerged in the process of adopting strategies to control defaecation dysfunction; these difficulties confused participants with respect to symptom management and influenced the effectiveness of these strategies.

#### 3.3.1 Confusion arising from conflicting information

To manage defaecation dysfunction, participants obtained information support from a variety of sources. Some of the information, especially the information related to diet, was contradictory, making participants feel confused. For example, one participant reported: “I asked the doctor how to manage the bowel symptoms, and he just told me there was no need to pay much attention to manage them because these symptoms would recover as time went by. But many other patients told me they tried to control them by themselves and some methods seemed work. What should I do?” (C29)

#### 3.3.2 Overreliance on personal intuition

The selection of strategies used for controlling defaecation dysfunction sometimes relied more on patients’ past experience than instructions from doctors, even though patients’ intuitive understanding was not suitable for managing bowel symptoms. For example, one participant said, “Although doctors told me that foods rich in fiber were good for smooth defecation, like corn and celery, I thought these foods were so stiff that they may hurt my bowel or they may damage the wound in my bowel. I have never eaten them during these months” (C19).

Some participants considered life experience passed down from generation to generation to be more reliable either for managing bowel symptoms or for decreasing the possibility of recurrence. One participant said, “The old people in our family always told us the Chinese chives were a stimulating food and that was also the rule from our old ancestors. I believed it without any suspicion, and it was forbidden in my diet” (C15).

#### 3.3.3 Confusion caused by the different requirements for treatment and controlling defaecation dysfunction

Some methods like controlling the total amount of foods at each meal and avoiding certain food, were used to relieve bowel symptoms by patients, however, these actions were not consistent with the requirement of obtaining sufficient nutrients while taking necessary precautions.

Some participants who were undergoing chemotherapy treatment were told to eat more nutritious foods; however, some of these foods could aggravate bowel symptoms. Participants needed to choose which requirement should be prioritized. Most of them chose to sacrifice the management of defaecation dysfunction and take actions to successfully finish chemotherapy. One participant said, “The more food I ate, the more frequent the defecation would be, and the anus would be more painful, but I also knew if I didn’t eat enough food then I couldn’t stand the chemotherapy anymore. That would be not good for my recovery. So I told the doctor once the chemotherapy was over, I won’t eat so much food anymore” (C13).

#### 3.3.4 Lack of self-discipline

In the interviews, dietary modification was reported to be an important method to control defaecation dysfunction. Participants realized that if they wanted to control their defaecation dysfunction, they had to give up some old dietary habits. As they sought to change their prior dietary habits, self-discipline was reported to obviously affect dietary behaviour. However, a lack of self-discipline was reported to influence dietary changes. For example, one participant reported, “doctors told me to eat more vegetables and fruits in my daily life, I tried my best to do that at the beginning after surgery, but I really don’t like eating vegetables, so I gradually quit nowadays.”(C26).

#### 3.3.5 Constraints related to objective social and family factors

The living environment and cooking habits of family members were considered objective factors influencing the self-management of defaecation dysfunction. The control of defaecation dysfunction could be constrained by these objective factors, especially because they could make changing eating behaviours difficult. For example, a participant said, “Recently, I was taking meals at my relative’s home, and she prepared many dishes including too much meat. I couldn’t blame her because of her kindness, but I did not want to eat so much fatty food in case it caused more frequent defecation” (C24).

The management of defaecation dysfunction was also constrained by social activities, such as eating meals with workmates. For example, a participant said, “my workmates often ordered so much dishes that I had eaten too much food unconsciously. I found that once I took meal with workmates at restaurant, I had to defecate more frequently the next day.” (C16).

## 4. Discussion

This study deeply explored the experience of controlling defaecation dysfunction among patients with rectal cancer following sphincter-saving surgery. The interview guide was designed according to symptom management theory (SMT), and the data collected on the self-management of bowel symptoms was therefore comprehensive. Several questions in the interviews were related to the “experience of symptoms” part of SMT, such as “Is there any defecation dysfunction existing after surgery?”, “What are the most distressing bowel symptoms?” and “How do symptoms influence your daily life?”. Questions about the “symptom management strategies” included “How do you cope with defecation dysfunction?”, “What factors do you think could influence your bowel symptoms? How do you deal with these factors?”, “How do you find methods to manage your bowel symptoms?” and “What do you think is the most difficult part of managing these symptoms and could you describe it in detail?” Questions related to “symptom outcomes” included “Do you think these bowel symptoms could be relieved by your management?” and “Who do you most want to give you suggestions and assistance about managing bowel symptoms and why?”

According to the findings in this study, defaecation dysfunction imposes a heavy burden on patients’ daily lives after sphincter-saving surgery. Defaecation urgency, leakage of stool and mixture of the stool and urine or flatus were the most troublesome symptoms during the first three months, and these contributed to the feeling of being “out of control”. This finding was also generally consistent with the literature from other countries, in which stool continence was noted to be most common just after surgery. (Hou et al.,^2017^; Keane et al.,^2017^; Emmertsen et al.,^2013^; Pietrzak et al.,^2007^). Another common symptom during the first three months was defaecating at night, and defaecating every two hours at night was a serious situation depriving the participants of sleep, which made them sleepy during the daytime. Compared to data in previous studies, this symptom seemed to be more significant. This symptom seemed to be more significant in this study than in previous studies. This might be due to the different evaluation tools used in the other studies or different emphases. (Hou et al.,^2017^).

Although bowel symptoms were common and severe, the control methods used by the participants varied and relied more on their own experience than evidence-based instructions. Self-management was the main strategy adopted by participants in this study, which was in line with several previous studies.(Nikoletti et al.,^2008^; Taylor & Bradshaw,^2015^).Self-management strategies in this study included dietary moderation, usage of pads, changes in social activity, suitable exercise and psychological moderation. Nearly half of the participants could not find an effective method for managing bowel problems because the discovery of the most suitable method depended on multiple factors, including their intuition, personality, education level, life habits, living environment and belief in using moderation to manage their symptoms. This seems to have been the reality of self-management among this group of patients, in line with previous studies. Desnoo published a qualitative study in which most participants described difficulty managing their symptoms, and the control strategies were not as effective as they were supposed to be. However, there was no in-depth analysis of why these difficulties existed in that study. (Desnoo & Faithfull,^2006^).

Among all the self-management methods, diet moderation was the most common approach noted by participants in this study. However, most of the participants sought to discover the optimal dietary pattern by trial and error, which involved much uncertainty about whether or how long they could successfully achieve suitable dietary moderation. These findings echo the trial-and-error strategy noted in previous studies (Sun et al.,^2015^). As a result, even though most participants changed their diet, the relationship between the types of food they ate and their defaecation patterns, according to some of them, was just conjecture. One of the most important reasons for this uncertainty about the dietary factor was that participants’ judgements were based on their own life experience or guesses and not informed by science. Further research might need to focus on providing scientific guidelines for participants to promote self-management. During dietary modification, traditional Chinese culture was an important factor influencing food selection. Participants preferred to avoid foods with a stimulating or cold nature because they may not be good for the recovery of bowel function. However, whether food choice based on traditional Chinese culture is a scientific way to control defaecation dysfunction still needs to be further discussed.

Exercise was shown here to play an unignorable role in the self-management of defaecation. However, the relationships noted by different participants were not consistent. The influence of exercise in this study was more marked than it was in the research conducted by Hou et al, in which exercise was referred to only as a way of regulating mood. Regarding the uncertainty of the specific effect of exercise on bowel symptoms, more studies on and more attention to this topic are needed.

According to the interviews, scientific guidelines for coping with bowel symptoms physiologically and psychologically are scant. Our patients received some instructions before discharge from the hospital, but the content of these instructions did not directly target the management of bowel symptoms. After they went home, there were almost no follow-up instructions, except for the consultation during the clinical re-examination, and that was the only chance participants had to ask for guidelines about how to control their symptoms. The instructions they received were limited, which was confirmed in other studies. L. DESNOO also found that a lack of support information was an obvious barrier, even though it was important(Desnoo & Faithfull,^2006^). Like Nikoletti’s subjects, the participants in this study thought patients needed more specific information about the function of every food and how to identify unsuitable foods (Nikoletti et al.,^2008^). Regarding the timing of receiving evidence-based guidance, for most participants, receiving guidance before discharge and during the recovery process was most desirable. This result contrasts with previous research, in which the need for information was greatest at diagnosis and at discharge. (Broughton et al.,^2004^). A possible reason for this difference was that our study focused mainly on the long-term recovery process, especially defaecation recovery, rather than the perioperative period.

The most suitable person to provide information was either the doctor or the nurse, according to our participants, and most participants did not emphasize whom they needed more. In other studies, there were no consistent views on this matter. Nikoletti surveyed patients’ information needs and showed that 46.2% of the participants named the doctor as the most important information resource, while only 21.2% of them named the nurse. A study from England conducted by Broughton found that professional nurses were the first choice for providing written guidelines and information support. In the future, more studies need to be carried out to make self-management strategies more rational and scientific.(Nikoletti et al.,^2008^)

Several limitations were exited in this study. First, participants were more likely to join in this study if they cared more about their bowel symptoms and health status. Participants were more likely to refuse to join in interviews if they gave more attention to the treatment after surgery than their own bowel symptoms. Second, there was a very small proportion of participants that were over 75 years old, and all patients came from the same tertiary hospital and this might reduce the representativeness of sample. Patients from various hospitals in different areas and patients with a larger age span are needed to recruited in following studies, in order to further enhance the diversity of participants.

## 5. Conclusions

The existing methods for the management of bowel symptoms were further explored from the perspective of participants in this study, but some barriers and difficulties exist in the process of controlling defaecation dysfunction, requiring much more attention from healthcare providers. There is a strong demand for systematic and scientific guidelines for the self-management of defaecation dysfunction. This study adds unique knowledge to the current information both on the specific deficiencies in the management of bowel symptoms and on possible ways to improve the management status.
